# The therapeutic effect of clinical trials: understanding placebo response rates in clinical trials – A secondary analysis

**DOI:** 10.1186/1471-2288-5-26

**Published:** 2005-08-18

**Authors:** Harald Walach, Catarina Sadaghiani, Cornelia Dehm, Dick Bierman

**Affiliations:** 1 University Hospital Freiburg, Brsg., Institute of Environmental Medicine and Hospital Epidemiology, Germany; 2 Samueli Institute, European Office, Universitatsklinikum Freiburg, Institut für Umweltmedizin und Krankenhaushygrene, Kugstether Strasse 55, 79106 Freiburg, Germany; 3 University of Freiburg, Brsg., Institute of Psychology, Germany; 4 University College Northampton, School of Social Sciences, UK; 5 University of Amsterdam, Department of Psychology, Netherlands

## Abstract

**Background and purpose:**

Placebo response rates in clinical trials vary considerably and are observed frequently. For new drugs it can be difficult to prove effectiveness superior to placebo. It is unclear what contributes to improvement in the placebo groups. We wanted to clarify, what elements of clinical trials determine placebo variability.

**Methods:**

We analysed a representative sample of 141 published long-term trials (randomized, double-blind, placebo-controlled; duration > 12 weeks) to find out what study characteristics predict placebo response rates in various diseases. Correlational and regression analyses with study characteristics and placebo response rates were carried out.

**Results:**

We found a high and significant correlation between placebo and treatment response rate across diseases (r = .78; p < .001). A multiple regression model explained 79% of the variance in placebo variability (F = 59.7; p < 0.0001). Significant predictors are, among others, the duration of the study (beta = .31), the quality of the study (beta = .18), the fact whether a study is a prevention trial (beta = .44), whether dropouts have been documented (beta = -.20), or whether additional treatments have been documented (beta = -.17). Healing rates with placebo are lower in the following diagnoses; neoplasms (beta = -.21), nervous diseases (beta = -.10), substance abuse (beta = -.14). Without prevention trials the amount of variance explained is 42%.

**Conclusion:**

Medication response rates and placebo response rates in clinical trials are highly correlated. Trial characteristics can explain some portion of the variance in placebo healing rates in RCTs. Placebo response in trials is only partially due to methodological artefacts and only partially dependent on the diagnoses treated.

## Background

Randomized controlled clinical trials (RCTs) usually employ a placebo control group to control for non-specific effects of therapy [1]. These effects comprise, among others, statistical regression to the mean, measurement artifacts, the natural course of diseases [2-4]. and true healing or improvement due to psychological or non-specific factors of therapy [5,6]. Thus, placebo response rates in clinical trials are always a mix of what has been termed true and false placebo effect [7]. True placebo effects have recently been defined as therapeutic effects due to the meaning of an intervention for a patient [8]. Some argue against the usage of the term "placebo effect" for such positive non-specific treatment effects, and observe that it is not possible to determine true placebo response rates in controlled clinical trials, since a natural history control group is lacking [9]. An analysis of three-armed trials with a natural-history control group in addition to treatment and placebo groups found no significant effect different from natural history for dichotomous outcomes, but a significant effect size of d = -.28 for continuos measures, which reflects mostly the placebo effect in pain [10]. This latter study, however, excluded a lot of high quality evidence from non-clinical, experimental studies, from non-pharmacological studies, from the psychological literature and older trials which were not randomized but are still very suggestive of clinical effects of placebos. If that material is also taken into account it seems difficult to dismiss a placebo effect in clinical trials outright [11]. A recent re-analysis of these data classifying studies into those that tried to maximize placebo effects and into trials that used placebo only as a control procedure found a large effect size of d = .95 for studies maximizing placebo effects, which was significantly different from studies using placebo as control (d = .15) [12]. It seems worthwhile, therefore, to try to understand what elements placebo response rates in randomized, placebo-controlled clinical trials (RCTs) are comprised of.

Although it would be ideal to launch a series of dismantling studies within the context of *clinical *trials to disentangle components of placebo response rates in RCTs, this is difficult. While in an experimental context many efforts have been presented recently, within a clinical context researchers, with a few exceptions [13,14]., seem reluctant to pursue the issue. A possible, albeit less reliable, route is the indirect study through secondary analysis of published trials. By using a non-selected sample of RCTs and correlating formal and informal study characteristics with the placebo response rate in these trials we tried to understand which characteristics of studies contribute to placebo response rates in RCTs.

In a previous secondary analysis of 26 RCTs with treatment duration of more than 12 weeks we found a significant correlation of r = .59 between placebo response rate and response rate in treatment groups [15]. Using a similar approach, Kirsch and Sapirstein [16] found an even higher correlation between treatment and placebo groups in trials of antidepressants (r = .90). When comparing these results with psychotherapy studies which used wait-list controls they estimated the specific treatment effect in those antidepressant trials to be 25% of the whole treatment effect. A more recent analysis of unpublished FDA licensing data of new antidepressant drugs, selective serotonin re-uptake inhibitors, corroborates these findings showing that 82% of the drug effect is duplicated by placebo, although the study arms are not significantly correlated [17]. Since the data of these study are from licensing studies which are proprietary and have not been subjected to peer review, it is difficult to know what to make of this information.

We decided to replicate and enlarge these findings by conducting another secondary analysis on a larger set of studies with a more heterogeneous set of diseases. Placebo response rates in RCTs might vary as a function of the disease studied, due to methodological artifacts because of low methodological study quality, as a function of time or due to other characteristics of a study. Thus, we retrieved a sample of unselected long-term trials. We coded several study characteristics and correlated them with the placebo response rate. We also operationalized study quality to find out whether placebo response rate is partially an artifact due to variation in quality. Our question was: Is the placebo response rate in RCTs dependent on time, study quality or other formal and informal study characteristics? In order to answer this question we conducted a systematic secondary analysis of published trial data.

## Methods

### Literature search and trial inclusion criteria

We searched the medical literature from 1992 to 1997 (MEDLINE, Cochrane Library) in order to find high quality long-term clinical trials. We also used existing meta-analyses in order to locate appropriate studies. Search terms were "placebo*", "double-blind*", "RCT*", "meta-analysis", "long*", "*studies", "treatment outcome". These search terms were combined with different strategies in order to optimize the results. We also handtracked the reference lists of existing studies and meta-analyses. Furthermore, we handsearched volume 1997 of main stream journals (Lancet, New England Journal of Medicine, British Medical Journal, Journal of the American Medical Association) as well as other leading medical journals. We also screened *Journal Watch*, Newsletter of the New England Journal of medicine, and *AIDS Clinical Care*.

Criteria for exclusion and inclusion of trials were formulated in a study protocol in advance.

All studies had to:

1 have a double blind placebo controlled randomized design

2 treat ill adults

3 provide a treatment of at least 12 weeks' duration

4 deal with a medical intervention with placebo control group

5 give sufficient information for to have outcome rates calculated for both groups

6 cover the period from 1992 to 1997.

### Data extraction

All data were extracted from the report of each trial with the use of a pretested form and entered into a spreadsheet. Formal characteristics were coded: (1) disease treated (ICD-10 diagnosis according to the first and second level); (2) duration of study; (3) is it a multi-center study (4) attrition rate (number of drop-outs in each group); (5) is the statistical evaluation of the study results done according to intent-to-treat analysis; (6) rating of study quality; (7) 'improvement' = response rate with placebo and medical treatment in a manner similar to [18] (i.e. % patients improved according to main outcome parameter mentioned in the study), or alternatively, in prevention trials, the number of patients (%) without event (e.g., cardiac) or worsening of the condition being under investigation (e.g., dementia);

### Quality rating

The rating of study quality is notoriously difficult [19]. Although the Jadad-score seems to be accepted since empirical validation studies exist [20-22], recent data also cast doubt on its usefulness [23]. Therefore, we followed recommendations to use a checklist adapted to the study question [19]. Our quality rating based on the one proposed by Detsky [24] and the Cochrane criteria [25] comprised the following items:

1. Description of inclusion and exclusion criteria?

2. Randomisation

3. Allocation concealment

4. Documentation of undesired events

5. Double-blinding of doctor, patient, and evaluator

6. Concomitant treatments

7. Description of statistical methods

8. Predefinition of outcome criteria

9. Documentation of patients lost to follow-up

Items 1, 4, 7-9 were answered in a yes-no format, items 2, 3, 5 and 6 were answered as "yes, and adequate", "yes, only mentioned", "no".

The coding of study quality was conducted by 2 of the authors with a subset of 20 studies rated jointly. A coding manual defined how to code for different aspects of study quality. The calculation of inter-rater reliability as intra-class correlation coefficient [26,27] showed sufficient consistency (r = .77); the widely used coefficient kappa was not applicable in our case due to the structure of the data matrix.

### Additional questionnaire information

Research on placebo effects normally starts from the presumption that placebo effects are due to instructions and contexts of the study, which induce expectations and other cognitions in patients as well as in physicians. In turn, these expectations give rise to biological processes and physical changes.

To test this hypothesis we constructed a questionnaire asking for additional information (e.g., amount of time spent with patients, amount of effort invested in the trial, indications of unblinding, expectations during the study). Our question was: is there a correlation between surrogate measures for expectancies of the principal investigators and the response rates.

For a subset of studies with recent publication dates we contacted the original researchers to elicit more information about study characteristics which are not normally reported in publications. We faxed or e-mailed a standardized questionnaire to those investigators who had responded positively to initial telephone requests. This questionnaire asked for informal addditional information. Four questions referred to organisational aspects of the trial (who initiated and financed the study?). Seven questions referred to possible aspects of methodological quality like unblinding, four questions were about time and intensity of contacts between the nursing staff, researchers and patients, and eleven questions referred to the attitude of the principal investigators towards the study, the study result and their expectations during the study. The questionnaire was originally in German, translated into English by a native speaker, who was fluent in German, and retranslated by a German native speaker fluent in English.

### Statistical evaluation

Diagnosis according to ICD 10 classification (first 2 descriptive levels) was converted into dummy variables (1,0-coding). All entered data were checked twice for plausibility and correctness by two independent persons. Descriptive information was taken from the study reports. The rating of study quality was added to a single unweighted quality score, after inspecting correlational patterns on single-item level. Improvement rate with treatment and placebo was defined as proportion of improved patients in relation to all patients treated within this group. Normally, this is reported in the original publications, or else it was calculated. Studies with more than two study arms were treated as two different studies, if separate placebo groups were employed. In dose finding studies with more than one treatment arm we always used the treatment group with the highest efficacy.

Data of the investigators' questionnaire was used on a single item level. Additionally, we formed different indices out of the items of the questionnaire which were meant to reflect "high involvement" (e.g. time, personal effort), high expectation or high importance of a study by grouping items according to their topic.

We calculated first order correlations of formal and informal study characteristics with placebo response rates. Based on these results and on our previous finding we formulated parsimonious regression models weighted by study size (n-3) as recommended in [28] to clarify which variables contribute to placebo response rates. In order to minimize capitalization of chance we only used theoretically interesting variables, those with significant first order correlations, and those that seemed promising after a first stepwise hierarchical model. In a second step we took out all non-significant predictors and entered all variables in the hypothesized sequence of importance in a forced regression model. Residuals were checked for possible non-linearity.

## Results

**Figure 1:**
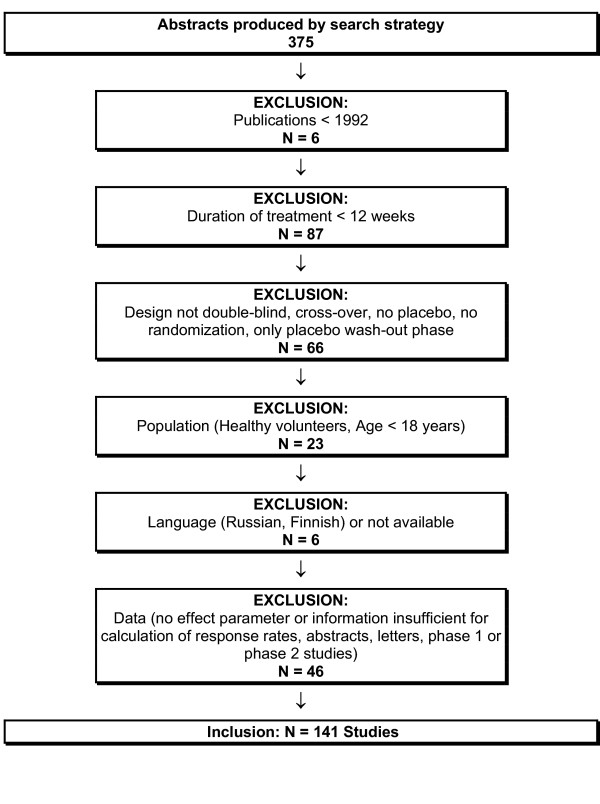
Flowchart of study inclusion.

Our search strategy produced after initial screening for sensitivity and specificity in its definite version 375 studies. The abstracts were screened for inclusion criteria. These were fulfilled by 141 studies. Figure 1 gives details about inclusion of trials.

Table 1 gives the number of studies according to ICD categories.

**Table 1:** Number of studies according to ICD categories

ICD-10 Category	Disease category	No of studies
A, B	Infectious and parasitical diseases	4
C	Neoplasms (Tumours)	5
E	Endocrine, nutritional and metabolic diseases	8
	Psychological and behavioural disorders	
F0	Organical, including symptomatical psychological disorders	4
F1	Psychol. and behav. disorders due to psychotropic substance abuse	22
F3	Affective disorders	13
F4	Neurotic, stress and somatoform disorders	12
F5	Behav. abnormalities with somatic disorders	5
F6	Personality and behavioural disorders	1
F8	Developmental disorders	1
G	Nervous diseases	14
	Diseases of circulatory system	
I10/11	High blood pressure	4
I20-I52	Ischemic and other forms of heart disease	21
I6/7	Cerebrovascular and other peripheral vascular diseases	2
J4	Pulmonary diseases	2
K	Digestive diseases	6
L	Diseases of the skin	1
M	Diseases of the muscular skeletal system and of connective tissue	9
N	Diseases of the urogenital system	3
T8	Trauma, intoxications and other consequences of extraneous causes	2
Y4	Extraneous causes of morbidity and mortality	2
	Number of all included studies	N = 141

Since in some studies there were multiple arms of treatment and control groups the basis for the statistical calculations is N = 153 independent combinations of placebo and treatment effects. Table 2 gives the significant first order correlations between the relevant variables of study characteristics.

**Table 2:** First order correlations between study characteristics (Pearson), N = 153

	%_T	%_P	DUR.	DIA_E	DIA_F0	DIA_F1	DIA_F4	DIA_G	DIA_ I1	DIA_ I2	PREV	INDEXQR
%_T	1,00	,78***	,29***	,01	-,14	-,33***	,01	-,31***	,14	,37***	,49***	,03
%_P		1,00	,41***	,07	-,16*	-,19*	-,11	-,30***	,12	,45***	,59***	,08
DUR.			1,00	,26**	-,08	-,18*	-,18*	-,16	,41***	,25**	,40***	,12
DIA_E				1,00	-,04	-,11	-,07	-,07	-,04	-,10	,25**	-,15
DIA_F0					1,00	-,07	-,05	-,05	-,03	-,07	,05	-,05
DIA_F1						1,00	-,13	-,14	-,08	-,19*	-,27**	-,03
DIA_F4							1,00	-,09	-,05	-,12	-,22**	-,02
DIA_G								1,00	-,06	-,13	-,24**	-,06
DIA_I1									1,00	-,08	,01	,05
DIA_I2										1,00	,54***	,14
PREV											1,00	,11
INDEXQR												1,00

*) p < .05 **) p < .01 ***) p < .001

Legend: %_T Percent of patients improved with treatment

%_P Percent of patients improved with placebo

DUR. Duration of study in months

DIA_E: Diabetes, other diseases of secretory glands and metabolism

DIA_F0: Dementia

DIA_F1: Behavioural disorders due to psychotropic substances

DIA_F4: Panic disorders

DIA_G: Epilepsy

DIA_I1: High blood pressure

DIA_I2: Ischemic and other forms of heart disease

PREV. Studies with preventive targets

INDEXQR Quality index

**Figure 2:**
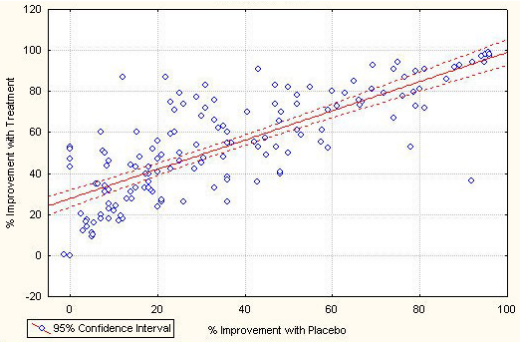
Scatterplot of response rate with drug and placebo for all studies

Improvement rates in placebo groups were significantly correlated with improvement rates in the treatment groups (r = .78; Fig. 2), and with duration of study (r = .41). If only the subset of 97 therapeutic trials excluding prevention studies are analysed, the correlation remains significant (r = .61). Placebo improvement rates are lower in trials of behavioral disorders due to psychotropic substances (r = -.19; recall that disease categories were dummy coded as 1: belonging to category; 0: not belonging to category), in dementia (r = -.16) and anti-epileptic trials (r = -.30). They are higher in prevention studies (r = .59) and trials of ischemic and other forms of heart disease (r = .45). They are unrelated to other forms of diagnosis and most notably unrelated to the quality of the trials, on single item level as well as on the index level, when considered as zero-order correlations. Nearly the same patterns of correlations can be found for the improvement rates with treatment.

We checked in a regression model for the predictive power of the combined variables. A significant regression model emerged that was able to predict nearly 80% of the variability of response in the placebo group (adjusted R^2 ^= .794; F _10/142 _= 59,74; p < 0.0001). This is presented in table 3. Main predictors were from two categories: methodological and diagnostic. Placebo response rates were larger for studies with longer study duration (beta = 0.31), for prevention trials (beta = 0.44), for multicentre trials (beta = 0.13) and for studies with better methodological quality (beta = 0.18). The questions whether dropouts (beta = -0.17) and additional treatments (beta = -.21) were described, were the important methodological variables. Therapeutic effects in placebo groups were smaller for trials of antitumor agents (beta = -0.21), studies in dementia (beta = -0.12), in substance withdrawal studies (beta = -0.14) and in studies of nervous diseases, mainly anti-epileptic trials (beta = -0.10).

**Table 3:** Regression model predicting variability in response rates in placebo groups of clinical trials; n = 153 studies/comparisons; formal and diagnostic variables (ICD coding)

	Beta	t (DFs = 142)	p-value
Constant		2.07	0.04
Duration in months	0.31	6.17	<0.000001
Multicenter trial	0.13	3.04	0.003
Quality Index	0.18	3.44	0.0008
Neoplasms (C)	-0.21	-5.48	<0.000001
Organical psychological disorders (F0)	-0.12	-3.24	0.001
Disorders due to substance abuse (F1)	-0.14	-2.97	0.003
Nervous diseases (G)	-0.10	-2.55	0.01
Prevention trial	0.44	7.94	<0.000001
Quality rating: Additional treatment described?	-0.21	-4.34	0.00003
Quality rating: Dropouts described?	-0.16	-3.33	0.001

Table 4 presents a subsidiary analysis for those 97 studies, which were not prevention trials. This regression model was also highly significant and explained 42% of the variance in placebo variability (adjusted R^2 ^= .426; F 5/91 = 15.22; p < 0.0001). Main predictors in this model were five diagnostic categories, with studies on substance withdrawal (beta = -0.31) and on nervous diseases/dementia (beta = -.0.20) showing lower placebo response rates, and studies in affective disorders, i.e. antidepressant and anxiolytic trials, (beta = 0.37), studies in digestive diseases, mostly inflammatory bowel conditions (beta = 0.29) and studies in urogenital diseases (beta = 0.16) showing better placebo treatment rates. Most notably, methodological quality did not contribute to the model.

**Table 4:** Regression model predicting variability in response rates in placebo groups of clinical trials; n = 97 Studies, with prevention trials excluded; formal and diagnostic variables (ICD Coding); italics: different from full model

	Beta	t (DFs = 91)	p-value
Constant		10.3	<0.000001
Disorders due to substance abuse (F1)	-0.31	-3.57	0.0006
*Affective disorders (F3)*	*0.37*	*4.49*	*0.00002*
Nervous diseases (G)	-0.20	-2.40	0.018
*Digestive diseases (K)*	*0.29*	*3.65*	*0.0004*
*Diseases of the urigenital system (N)*	*0.16*	*2.09*	*0.039*

### Additional questionnaire information

Principal investigators of 57 recently published studies were asked for retrospective additional information. 50 of those answered our request (87%), and 44 supplied sufficient data. There is only one significant first order correlation out of all items. Apart from the correlation between placebo and treatment response rates there is no significant and substantial correlation except for chance fluctuations. But we found evidence for unblinding in 30% of the 44 responses.

### Monte Carlo simulation

One of the possible sources of the correlation between placebo and verum effect might be publication bias, especially when there is no significant difference between the placebo and verum condition. In order to explore this potential source of the correlation we simulated the situation of publication bias by assuming that only trials were published where the differential effect had a chance probability of p < 0.01

The simulation parameters used were:

a) mean effect size of the placebo: ESp

b) mean effect size of the verum: ESv

c) Number of subject in a trial: Nss

d) Number of experiments per effect: Nexp

**Figure 3:**
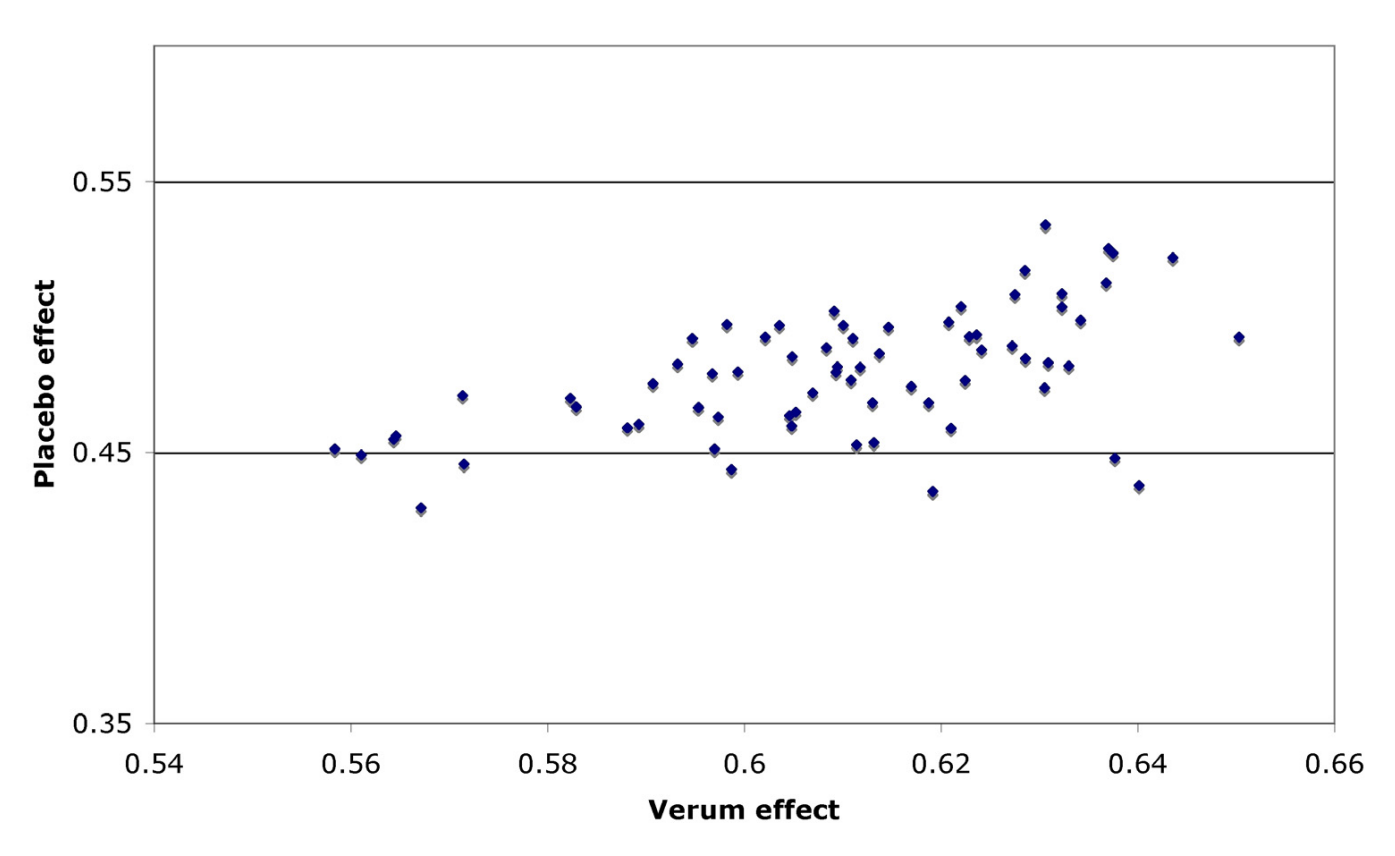
Scatterplot of results of simulation of the effect of publication bias. Simulation parameters were: Esp = 0.5, ESv = 0.51 - 0.60 in steps of 0.01, Nexp = 50, Nss = 100. All non-significant trials removed

If no publication bias is assumed, there is no correlation between verum and placebo effect sizes. However, there were significant correlations when a publication bias was introduced. This was the case for each reasonable choice of simulation parameters. In Figure 3 a scatterplot for published placebo and verum effect sizes are given for a typical choice of simulation parameters. The correlation obtained is r = 0.63, close to the true correlation. In other words publication bias could, in principle, explain nearly all variance, assuming that all non-significant studies are suppressed. It should be noted, however, that the filedrawer for this particular simulation contains 86% of all experimental results. It is improbable that so many trials would remain unpublished.

## Discussion

This secondary analysis was motivated by the attempt to understand what influences placebo response rates in RCTs. We found a rather strong correlation between improvement rates with placebo and treatment of r = .78, which explains roughly 60% of the variance. The magnitude of the correlation drops somewhat to r = .61, explaining 37% of the variance, if only therapeutic trials are considered. This result is in concordance with our own and other previous findings [15,17,29]. It is incidentally backed by an early result reported by Evans [30]. Although Evans' finding has been explained as a result of the skewed distribution of the underlying binary data [31], this explanation does not apply to our data, which followed a Gaussian distribution.

This high correlation should not come as a surprise, as in clinical trials a certain basic effect, thought to be covered by the placebo control group, is compared with this same basic effect plus some specific element of pharmacologic intervention. That is, effects in clinical trials are bound to be correlated. Indeed, if there were no intervention effects at all we would have a perfect correlation of r = 1.0. The fact that the correlation is not perfect is a sign that treatment and control groups behave differently. The fact that the correlation is so high and in fact explains 60% of the variance is a sign that the commonalities of factors in groups within trials is greater than their difference. In other words, non-specific treatment effects are more important than the specific ones. Thus we have once more corroborated a now growing body of evidence about the importance of non-specific treatment effects [14,32].

Natural history or cohort effects might be an explanation for the high correlation between treatment and placebo response. The correlations found in our study are suggestive of an overall small to moderate treatment effect reflecting in an imperfect correlation. Although a cohort effect is a possible explanation, we would like to point out that durations of trials are normally chosen according to the disease studied and to cover a time period which captures a relevant portion of natural fluctuation. For instance, depression maintenance trials are normally conducted over a time span long enough to make a recurrence in untreated patients likely, or dementia trials cover a period, where a progression of the disease is to be expected. Thus, natural history is partly covered by the differing trial durations of the studies analyzed. If natural history were the *only *factor reflected in the improvement rates of placebo groups, on average we should expect deteriorations or little improvement in placebo groups, because the diseases studied here are mostly long-term chronic diseases with little self-limiting tendencies. Across all studies, diseases and trial durations of a heterogeneous set of studies we should expect that cohort effects average out and the correlation between treatment and placebo improvement should tend towards zero [31]. In fact, our simulation produces a zero-correlation for many different scenarios of study outcomes, confirming this intuitive reasoning. Thus, there seem to be non-specific elements of treatment at work here that reflect in improvement rates in placebo groups which are grossly comparable to those in the treatment groups.

What is important from a differential point of view is, whether these non-specific elements of treatments, or the variability of responses in the placebo groups, can be further elucidated. Our regression analysis shows that a series of formal characteristics is responsible for this variability of therapeutic responses in placebo groups. The fact that this analysis can explain nearly 80% of the variance is a support for the hypothesis that a large part of those non-specific effects in randomised placebo controlled trials is due to formal characteristics. The effect in placebo control groups is higher in studies with a longer duration and in prevention trials. Prevention trials are normally also longer in duration. In fact this effect of duration of trial vanishes when prevention trials are taken out of the analysis (compare Tables 3 and 4). This strong effect of prevention trials is worth considering. It means that in such trials the event rate in the control group is smaller, i.e. the placebo healing rate higher, than in comparable curative trials. It may be the psychological focus on preventing an event that triggers effects different from placebos in curative trials. It may simply be the fact that in prevention trials medications, and placebos, are given for a very long time, sometimes over years, and hence expectations for maintainance of a comparatively healthy state are continuously reinforced. Or it may be easier to harness expectations for maintaining a state of comparative well-being than to use them for getting well again. This effect of prevention trials warrants a deeper scrutiny than can be provided by a retrospective analysis.

This predictive power of prevention trials is by far the strongest effect in our data base, which also explains partially the other elements: prevention trials are normally not only longer, but also larger and require the effort of many centres. Therefore they are in tendency better planned and have a slightly better methodology. These methodological factors disappear, when the analysis is repeated with prevention trials excluded. It is obvious that placebo variability is different for different diseases. In the full data-set trials of anti-tumor agents, of anti-epileptics, of anti-dementia drugs, and of substance withdrawal contribute to variability by exhibiting smaller placebo effects. However, this should not blind us to the fact that the beta-weights, which are correlation coefficients adjusted for the effects of all other variables, of these diagnostic variables are rather small. In the reduced data-set, without the prevention trials, improvement rates in placebo groups are higher in studies of affective disorders, which comprise mainly antidepressant and anxiolytic studies, in studies of anti-inflammatory agents in bowel diseases and in studies of urogenital diseases. Improvement rates are lower for substance withdrawal studies and anti-epileptics. The fact that the regression model in the reduced data-set explains only 42% of the variance and thus has approximately only half the explanatory power of the full set shows that other unknown factors are operative, besides spontaneous improvement rates in different diseases. Otherwise we would expect a stronger effect of the diagnostic categories in our regression model.

Our simulation runs brought one other explanatory option to the fore: publication bias. Under the assumption that only trials are published that prove the significance of the treatment over placebo and the non-significant trials are filed away, simply by this publication bias alone, a correlation can be obtained which is close to the one we observed empirically. Thus it is tempting to assume that the true reason for the high correlation between treatment and placebo improvement rates is, at least partially, publication bias. This points, once more, to the importance of publishing all evidence, not only positive studies. However, bearing in mind that the simulation produced a correlation of r = .63 under the precondition that *all *non-significant evidence, i.e. 87% of all evidence remained unpublished, publication bias cannot account for the whole correlation, but perhaps for a substantial amount.

Although not quite as obvious, one of the most important messages of this study is hidden behind what we have *not *found:

There is no sizeable effect of methodological quality apart from the one discussed above which is explained by the prevention trials. This means that placebo variability in clinical trials is not only due to methodological artifacts, as is sometimes suggested [2,3], at least as assessed by our scale.

Frequently it is proposed that placebo responses are due to heightened expectations of investigators and subsequently patients in trials. Since trials are conducted with more frequent and more intense contacts between doctors and patients, the argument would run, patients could form stronger expectations of improvement, which in turn could lead to response expectancies, which again would result in clinical improvement [33]. We could not test this hypothesis directly. But we did test it indirectly by asking principal investigators to give information on study characteristics not normally reported in publications, like amount of time spent with patients, amount of effort invested in a trial, importance of the trial for further funding, expectation of investigator and the like. None of these indirect operationalizations of heightened expectation and increased effort on part of the investigators showed any notable correlation with placebo response rates.

We found substantial evidence for unblinding of investigators in 30% of 44 trials with additional questionnaire data. Surprisingly enough, placebo response rate was *uncorrelated *with unblinding. Thus, patients in studies with indications for investigator unblinding had a higher improvement rate only in the treatment group, but not in the placebo group. If investigator opinion was a decisive factor in the creation of patient expectancies, we would expect a substantial negative correlation between unblinding and placebo response rate. Since unblinding occurred in 30% of the cases, we should have been able to see such a correlation if it had been present. It is obviously not an issue. Thus, informal characteristics in studies, reflecting a higher awareness and a stronger engagement of investigators and patients in clinical trials, do not seem to influence the amount of improvement in placebo groups. We submit that this part of our data is rather weak, since it is retrospective evidence. Nevertheless, it is a first empirical data set which could be followed up by prospective studies.

What other explanations or mechanisms could be responsible for this effect? One idea is well known and posits that clinical trials are in fact healing rituals with a strong non-specific effect [33]. The other invokes the new concept of generalized entanglement in systems.

### Trials as healing rituals

Treatment effects of mostly pharmacological interventions studied here do have some effect, but the effect is smaller than those of non-specific effects. It has been argued that therapeutic effects are meaning effects in general, with pharmacological or other medical interventions adding a plausibility factor that actually triggers this meaning effect [34]. A meaning effect would be an individual healing effect induced by the complex interaction between an individual, constructing meaning out of his or her medical situation, the medical system trying to intervene, physiological changes brought about by the interventions and psychological changes inferred by the individual and hence altered psychological states. These states have been documented as altered brain function [35-40] and hence it is plausible to assume that they can also affect complex medical conditions.

Such an interpretation could be supported by our data, since the non-specific elements are by far the more important ones, compared with the specific elements of treatment. Non-specific effects account for nearly 60% of the variance of all treatment effects. This is true for most disease categories and across a wide variety of interventions. This alone should be an intriguing result, stimulating more research effort into mechanisms and processes of unspecific effects of therapy in general and trials in particular.

Correlation effects as an instance of generalized entanglement:

According to a weaker and generalized version of quantum theory [41], applicable to different systems outside the realm of physics, entanglement, i.e. non-local correlations structurally similar to but factually different from quantum correlations, would be expected in any system with a global observable and a local observable that are complementary and do not commute; in addition they may have to share a common contextual history (D. Gernert, personal communication). A clinical, blinded trial is such a system by definition. The global observable is the blinding of the trial, the local observable is the actual allocation of patients to treatment groups. Global blinding and local definite allocation are complementary notions, and thus entanglement would be predicted, visible as a correlation across trials. The common context is given by the aim and the procedures of the trial. This hypothesis, which has received some support recently by experimental data in other areas [42], would have to be investigated further by means of direct experimentation. For the time being this is only a speculative possibility. However, should it bear out, it would have consequences both for study methodology and for clinical practice. On the one hand, it would be expected that medications show different, mostly stronger, effects in unblinded contexts, on the other hand it would follow that data from blinded trials are biased estimators of real effect sizes.

We would like to add some cautions:

It is difficult to draw firm conclusions about non-specific effects from two armed-trials [9]. This should be born in mind, when interpreting our data, since all our evidence and argument is indirect by default. Our retrospective questioning of principal investigators may be considered weak. While we agree that only a few investigators could be queried and retrospectively gathered evidence is far from compelling, there is no selection behind our sampling procedure but only temporary sequence. And among those selected we achieved a high return rate. Thus we are confident that we have at least given a small part of the picture reliably, and it goes without saying that this piece of our evidence can only be a hint for further research. It should be elucidated in prospective analyses, whether and how often unblinding is seen and how this affects outcome rates. It could be documented before knowledge of outcomes, how strong extra involvement and enthusiasm of doctors and investigators have been compared to conventional clinical practice. Until such prospective evidence is available, we have produced at least some estimators of trial effects and have not found convincing evidence for them.

## Conclusion

We conclude that the placebo response rate in controlled clinical trials is not due to methodological artifacts, to disease history alone or to circumstantial characteristics of studies, but seems to reflect a genuine improvement, unless one invokes publication bias for all negative studies. This improvement accounts for roughly 60% of the variance of all therapeutic gains across trials.

## Competing interests

The author(s) declare that they have no competing interests.

## Authors' contributions

Harald Walach devised the study, organised funding, supervised the study, participated in the final evaluation, wrote the manuscript and had part in the interpretation of the data.

Catarina Sadaghiani was a PhD-student on the project, conducted the study, collected, prepared and analysed the data, had part in the final evaluation and in the interpretation of the data.

Cornelia Dehm was a diploma student on the project, jointly supervised by HW and CS. She did the interview-part of the study and helped with interpretation.

Dick Bierman helped with data-analysis and interpretation and conducted the Monte-Carlo simulation.

## Pre-publication history

The pre-publication history for this paper can be accessed here:


